# The effect of *Lactobacillus fermentum* DALI02 in reducing the oxidative stress and inflammatory response induced by high-fat diet of rats

**DOI:** 10.1039/d0ra05694d

**Published:** 2020-09-16

**Authors:** Yujun Huang, Hengxian Qu, Dong Liu, Yunchao Wa, Jian Sang, Boxin Yin, Dawei Chen, Xia Chen, Ruixia Gu

**Affiliations:** College of Food Science and Technology, Yangzhou University Yangzhou Jiangsu 225127 China guruixia1963@163.com; Key Laboratory of Dairy Biotechnology and Safety Control Yangzhou Jiangsu 225127 China

## Abstract

A long-term high-fat diet (HFD) leads to significant oxidative stress in the body and induces inflammation. A preliminary evidence suggests a potential therapeutic utility of probiotics for this condition. To evaluate the potential effect of *Lactobacillus fermentum* DALI02 on the oxidative stress and inflammatory damage induced by HFD, we used a hyperlipidemic rat as a model fed with HFD. Results revealed that HFD induced a significant oxidative stress and inflammation. However, results reveal that *L. fermentum* DALI02, manifested a significant decrease in levels of malondialdehyde (MDA), tumor necrosis factor-α (TNF-α), interleukin-6 (IL-6) and resistin, while the catalase (CAT), total antioxidant capability (T-AOC), superoxide dismutase (SOD), glutathione peroxidase (GSH-Px) and adiponectin (ADPN) levels significantly increased. And it was dose-dependent that the effect of high dose groups with high viable count was particularly notable. The results suggest that *L. fermentum* DALI02 could alleviate oxidative stress and inflammation as it appeared to reduce lipid peroxidation and improved the lipid metabolism *in vivo*.

## Abstract 2

A long-term high-fat diet (HFD) leads to significant oxidative stress in the body and induces inflammation.

## Graphical abstract

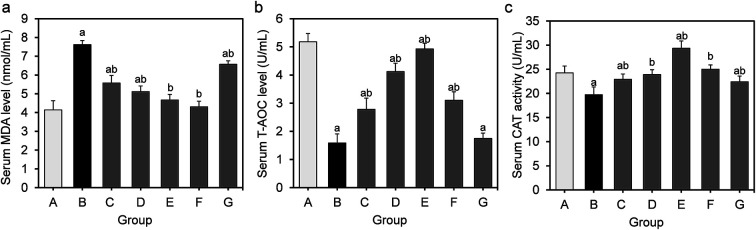

## 1 Introduction

Organisms exist in pro-oxidant and antioxidant homeostasis, but this balance, which is affected by various factors is not stable. The balance is broken by the accumulation of high levels of reactive oxygen species (ROS), which induces oxidative stress (OS) and damages DNA, proteins and lipids.^1^ Many human diseases including cancer, obesity, atherosclerosis and liver damage are correlated with OS.^2^ The irrational dietary structure is a common factor that leads to the oxidative stress.^3^ Studies have reported that a long-term HFD induced a significant oxidative stress in lipid metabolism-related organs such as the pancreas, liver and intestine,^4^ affecting the body's immune function and induced chronic inflammation.^5^ However, a sufficient antioxidant supply may help prevent the course of a disease^4^ and alleviate inflammatory response.^5^ Synthetic antioxidants such as butylated hydroxyanisole (BHA), butylated hydroxytoluene (BHT) and *n*-propyl gallate (PG) pose potential risks *in vivo*; their use in food is restricted or prohibited in some countries. Therefore, antioxidants from natural sources are much desirable.

Probiotics are living microorganisms, which confer health benefits on the host when administered in adequate amounts.^6^ Probiotics have proved to be beneficial in treating diarrhea, metabolic disorders, lowering blood pressure and serum cholesterol, and are safe as well.^7,8^ In recent years, many studies have demonstrated that some probiotics also have the antioxidant capacity. In addition, many researchers have confirmed that probiotics could also alleviate oxidative stress to some extent *in vivo via* the endotoxin and d-galactose-induced oxidative stress injury model,^9^ excessive intake of iron-induced oxidative stress model^10^ and alcohol-induced oxidative stress injury model.^11^ The antioxidative effect of probiotics may be an important mechanism involved in this function.^12^ However, there are only few studies on the effects of probiotic on the oxidative stress and inflammatory response induced by HFD. The HFD-related obesity is considered a result of chronic inflammation induced by intestinal microbiota.^13^ Hyperlipidemia is associated with the oxidative stress and inflammation.^4^ Tian^3^ showed that HFD caused a change in the intestinal flora, but the balance of the micro-ecological environment could be restored to some extent by the supplementation of probiotics, thus easing the oxidative stress induced by HFD. Esposito *et al.*^14^ reported that probiotics VSL # 3 could reduce liver oxidative stress and inflammatory response induced by HFD in young rats. However, the lack of research on the effects of forms and doses of probiotics on their function needs to be investigated.

*L. fermentum* DALI02 has been observed to possess a high survival rate in artificial gastric fluid, artificial intestinal fluid and bile salt, and has good cholesterol-reducing and antioxidant ability *in vitro*. Therefore, it is possible that *L. fermentum* DALI02 could relieve hyperlipidemia and help alleviate oxidative stress and inflammation. We tested this hypothesis on hyperlipidemia rats induced with high-fat diet and initially explored the mechanism of its effect.

## 2 Materials and methods

### 2.1 Microorganism and preparation of oral samples

*L. fermentum* DALI02 was cultured in sterile MRS broth (Difco, Detroit, Mich., USA) from the 3% inoculum with 18 h of incubation at 37 °C. Cell pellets were harvested at 5000 × *g* for 10 min, washed three times with PBS and then lyophilized (skimmed milk as the protective agent). The lyophilized powder was suspended in a physiological saline solution as a bacterial suspension and adjusted to different doses for the oral administration to rats. Then, the bacterial suspension was ultrasonicated for 10 min (100 W, working time/space time at 3 s/5 s) in ice bath to obtain bacterial lysates. The fermented milk was obtained by inoculating with 3% (v/v) of *L. fermentum* DALI02 in sterile 10% (w/v) skimmed milk at an initial population of approximately 10^6^ CFU mL^−1^, and incubated at 42 °C to curd and adjust cell numbers to 10^9^ CFU mL^−1^.

### 2.2 Animals and diet

Male and female Wistar rats, weighing 90–110 g of 3 week-old, were obtained from the Comparative Medicine Center of Yangzhou University and kept in an air-conditioned room maintained at 23 ± 2 °C and 55 ± 10% relative humidity under a 12 h light/dark cycle. The animals were allowed free access to food and water, and had one week to acclimatize to the laboratory environment. All animal procedures were performed in accordance with the Guidelines for Care and Use of Laboratory Animals of Yangzhou University and approved by the Animal Ethics Committee of Yangzhou University.

The composition of the basic experimental diet is shown in Table 1. The fat-rich diet consisted of the basic experimental diet (78.8%, w/w), egg yolk powder (10.0%), lard (10.0%), cholesterol (1.00%) and sodium cholate (0.2%).

**Table 1:** Compositions of the basic diet (%)

Ingredient	Content (%, w/w)
Wheat flour	20.0
Rice flour	10.0
Corn flour	20.0
Wheat bran	26.0
Soybean pieces	20.0
Fish meal	2.0
Bone flour	2.0

### 2.3 Experimental design

The experimental animals were randomly divided into 7 groups with 10 animals per group (named as A, B, C, D, E, F and G groups, respectively) after acclimatization for 1 week using the basic experimental diet. The group A was given a basic experimental diet, and other groups were given the high fat diet for 8 weeks. The experimental diet given was approximately 20 g/100 g BW per day. The hyperlipidemic rat model was induced by the consumption of high-fat diet for 4 weeks. From the fifth week, all rats received the following treatments by lavage. Groups A and B were the control group and the hyperlipidemic model group, respectively, treated with skim milk. Groups C, D and E were the treatment groups with different doses of bacterial suspension (skimmed milk) of *L. fermentum* DALI02 from low to high. Group F was treated with fermented milk of *L. fermentum* DALI02, and group G was treated with bacterial lysate (skim milk) of *L. fermentum* DALI02. The dose of all oral samples for each animal was calculated as 10 mL per kg BW per day for another 4 weeks after the hyperlipidemic rat model induction. Total viable CFU of groups C, D, E, F and G was 1 × 10^7^, 1 × 10^8^, 1 × 10^9^, 1 × 10^9^, and 1 × 10^9^ CFU mL^−1^, respectively. The animals were fasted for 12 h before being sacrificed.

### 2.4 Assay of blood lipid levels in the serum

After 2 and 4 weeks of feeding, rats in the basic diet group (A) and the high-fat diet group (B–G) were randomly selected for capillary blood sampling. Serum was collected by centrifugation at 3000 × *g* for 15 min and stored at −20 °C. Total cholesterol (TC), triglyceride (TG) and low density lipoprotein cholesterol (LDL-C) of the supernatant were detected according to standard procedures with assay kits (Nanjing Jiancheng Bioengineering Institute, Nanjing, China) using a fully automatic biochemical analyser (Hitachi, JAPAN).

### 2.5 Assay of MDA, T-AOC and CAT in serum

Blood was obtained from each group after 8 weeks. Serum was collected by centrifugation at 3000 × *g* for 15 min and stored at −20 °C. Contents of MDA, T-AOC and CAT were determined following standard manuals supplied with assay kits (Nanjing Jiancheng Biotechnology Institute, China). The content of MDA was determined by the thiobarbituric acid method. The T-AOC assay was performed using the ferric reducing-antioxidant power assay. The CAT activity was determined by measuring the intensity of a yellow complex formed by molybdate and hydrogen peroxide at 405 nm after ammonium molybdate was added to terminate hydrogen peroxide degradation, and was catalysed by catalase.

### 2.6 Assay of MDA, T-AOC, SOD and GSH-Px in liver homogenate

Liver was removed at the time of sacrifice. Ten percent of liver homogenates was obtained by homogenizing liver tissues in a cold physiological saline solution (4 °C). Consequently, liver homogenates were centrifuged at 3000 × *g* for 15 min at 4 °C, and the protein concentration, MDA, T-AOC, SOD and GSH-Px of the supernatant were detected according to standard procedures with the assay kits (Nanjing Jiancheng Bioengineering Institute, Nanjing, China). The SOD activity was measured by the xanthine oxidase method. The GSH-Px activity was determined by measuring the reduction of glutathione. The protein concentration was determined by the BCA protein assay.

### 2.7 Assay of MDA, T-AOC, GSH-Px, TNF-α, IL-6, ADPN and resistin in adipose homogenates

A part of the perirenal adipose tissue was put in cold physiological saline immediately and a tissue homogenate was prepared (10%, w/v). The adipose homogenates were centrifuged at 3000 × *g* for 15 min at 4 °C, and the supernatant was collected and stored at −20 °C until further analysis. The levels of MDA, T-AOC, GSH-Px in adipose homogenates were detected with the assay kits (Nanjing Jiancheng Bioengineering Institute, Nanjing, China). The contents of TNF-α and IL-6 were determined by the double antibody sandwich ELISA method with ELISA kits (Shanghai ExCell Biology, Inc, Shanghai, China), while the contents of ADPN and resistin were determined by the double antibody sandwich ELISA method with ELISA kits (Nanjing Jiancheng Bioengineering Institute, Nanjing, China).

### 2.8 Statistical analysis

Results were expressed as mean ± standard deviation (SD). Differences between groups were evaluated using one-way analysis of variance (ANOVA), followed by the Tukey test (SPSS for Windows Release 16.0, SPSS Inc., Chicago, IL, USA). The *P*-value of less than 0.05 was considered significant.

## 3 Results

### 3.1 Effects of high-fat diet on blood lipid levels in the serum of rats

The effects of high-fat diet on the TC, TG and LDL-C levels in serum of rats are shown in Table 2. After feeding for 4 weeks on a high-fat diet (compared with the control group), the TC, TG and LDL-C levels in serum of rat significantly increased (*P* < 0.05), indicating the successful establishment of a rat hyperlipidemia model at 4 weeks.

**Table 2:** Change in the serum lipid level of rats fed with basic and high-cholesterol food^a^

Group	Weeks (w)	TC (mmol L^−1^)	TG (mmol L^−1^)	LDL-C (mmol L^−1^)
A (*n* = 5)	2	1.88 ± 0.14^a^	0.80 ± 0.14^a^	0.42 ± 0.08^a^
	4	1.84 ± 0.17^a^	0.75 ± 0.10^a^	0.39 ± 0.06^a^
B–G (*n* = 12)	2	2.49 ± 0.18^b^	0.98 ± 0.18^ab^	0.85 ± 0.08^b^
	4	2.87 ± 0.12^b^	1.31 ± 0.17^b^	0.90 ± 0.07^b^

a Compared with the same column, different letters indicate significant differences (*P* < 0.05).

### 3.2 Effects of *L. fermentum* DALI02 on oxidative stress in the serum of rats

**Fig. 1:**
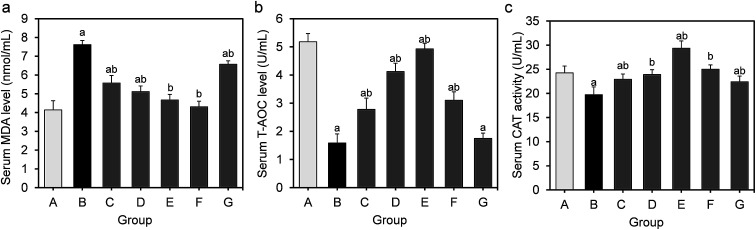
Effect of *L. fermentum* DALI02 supplementation on MDA, T-AOC, CAT levels in the serum of rats. (a) Serum MDA level. (b) Serum T-AOC level. (c) Serum CAT activity. ^a^*P* < 0.05 *vs.* group A (normal group); ^b^*P* < 0.05 *vs.* group B (model group).

The effects of feeding *L. fermentum* DALI02 on MDA, T-AOC, CAT activity levels in the serum are shown in Fig. 1. The results show a 1.8-fold increase in the serum MDA from the hyperlipidemic model group relative to the normal control group, while the activities of T-AOC and CAT significantly decreased (*P* < 0.05); it indicated that long-term HFD resulted in the development of oxidative stress in rats.

T-AOC and CAT were improved in serum by the *L. fermentum* DALI02 treatment and the improvement of T-AOC was dose-dependent. It was observed that the increase in the levels of T-AOC and CAT was higher than the normal level with the administration of the high-dose bacterial suspension. The probiotic treatment also decreased the level of MDA. There was a gradual decrease in the serum MDA level from low dose to high dose (groups C and D), and fermented milk treatment resulted in the lowest MDA level in serum. However, the cell lysates of *L. fermentum* DALI02 seemed to be less effective, and the levels of T-AOC were no statistically significant among groups B and G.

### 3.3 Effects of *L. fermentum* DALI02 on oxidative stress in the liver of rats

**Fig. 2:**
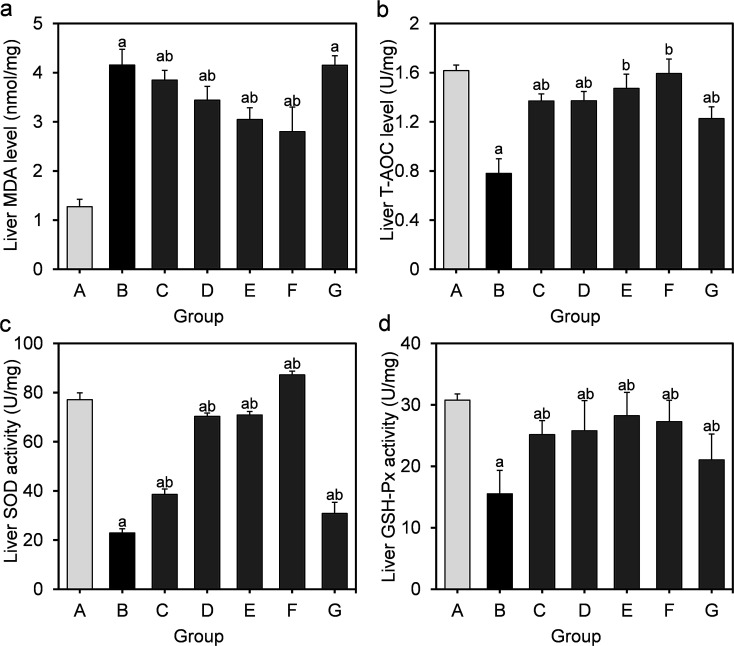
Effect of *L. fermentum* DALI02 supplementation on MDA, T-AOC, SOD and GSH-Px levels in the liver of rats. (a) Liver MDA level. (b) Liver T-AOC level. (c) Liver SOD activity. (d) Liver GSH-Px activity. ^a^*P* < 0.05 *vs.* group A (normal group); ^b^*P* < 0.05 *vs.* group B (model group).

Obesity induced by long-term HFD results in steatosis and increases oxidative stress in the liver. The effects of *L. fermentum* DALI02 on the level of MDA as well as the activities of the antioxidant enzymes in the liver are presented in Fig. 2. MDA levels in the model group were significantly higher than that of the normal control group (by a 3.3-fold increase), indicating the over-peroxidation of liver injury. At the same time, significantly lower liver T-AOC, SOD and GSH-Px activities were observed in rats in the model group relative to those in the normal control group. In the liver, supplementation of *L. fermentum* DALI02 significantly increased the activities of T-AOC, SOD and GSH-Px (*P* < 0.05), and group E and group F (namely high-dose bacterial suspension and fermented milk) showed the best results. However, dose-dependence was not obvious. The content of MDA was reduced by the addition of *L. fermentum* DALI02 as compared with the model group, and the fermented milk treatment resulted in the lowest MDA level. These results suggest that treatment with *L. fermentum* DALI02 could enhance the hepatic antioxidant status.

### 3.4 Effects of *L. fermentum* DALI02 on oxidative stress in adipose of rats

**Fig. 3:**
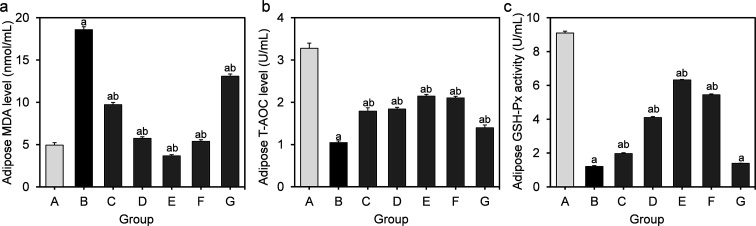
Effect of *L. fermentum* DALI02 supplementation on MDA, T-AOC and GSH-Px levels in the adipose of rats. (a) Adipose MDA level. (b) Adipose T-AOC level. (c) Adipose GSH-Px activity. ^a^*P* < 0.05 *vs.* group A (normal group); ^b^*P* < 0.05 *vs.* group B (model group).

The effects of *L. fermentum* DALI02 on the level of MDA as well as on activities of T-AOC and GSH-Px in the perirenal adipose are presented in Fig. 3. As compared with the normal control group, the MDA levels of the model group showed a significant 3.8-fold increase (*P* < 0.05), while the activities of T-AOC and GSH-Px significantly decreased (*P* < 0.05), indicating that the oxidative stress induced by HFD was also significant in adipose.

The higher levels of MDA were significantly reduced by the treatment with *L. fermentum* DALI02. There was a gradual decrease in the MDA level from low dose to high dose (groups C and D), and the group E even recovered to the normal level. There was a significant increase in the activities of T-AOC and GSH-Px in groups treated with *L. fermentum* DALI02, but the cell lysates of *L. fermentum* DALI02 seemed to be less effective in adipose, and there was no significant increase in the GSH-Px activity, as compared with the model group.

### 3.5 Effects of *L. fermentum* DALI02 on the adipocytokines of rats

**Fig. 4:**
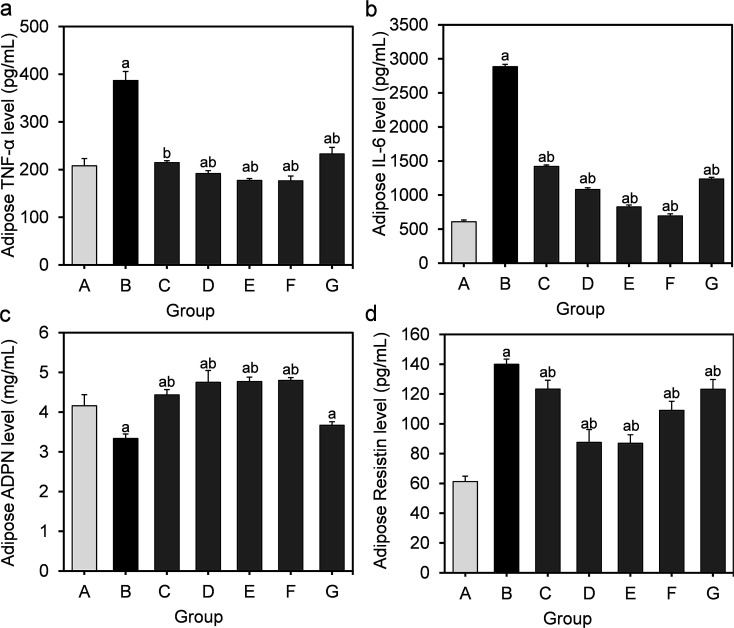
Effect of *L. fermentum* DALI02 supplementation on TNF-α, IL-6, ADPN and resistin levels in the adipose of rats. (a) Adipose TNF-α level. (b) Adipose IL-6 level. (c) Adipose ADPN level. (d) Adipose resistin level. ^a^*P* < 0.05 *vs.* group A (normal group); ^b^*P* < 0.05 *vs.* group B (model group).

To further assess the inflammation induced by HFD, we measured the TNF-α, IL-6, ADPN and resistin levels in the supernatants of adipose homogenates. It can be seen from Fig. 4 that the level of TNF-α, IL-6 and resistin in the model group markedly increased compared to those in the normal control group, while the ADPN level significantly decreased (*P* < 0.05).

Similarly, the supplementation of *L. fermentum* DALI02 evidently suppressed the production of TNF-α and IL-6 as well as stimulated the induction of ADPN. The effect of high and medium dose bacterial suspensions seemed to be better, as a significantly lower TNF-α, IL-6 and resistin levels were observed in rats relative to the model group, and the effect was dose dependent. We found a similar effect in the fermented milk group. However, the cell lysates of *L. fermentum* DALI02 seemed less effective, as compared with the model group, and there was a slight increase in the ADPN level but not significant.

## 4 Discussion

The purpose of this study was to evaluate the protective effect of supplementation of foods containing *L. fermentum* DALI02 on oxidative stress and inflammatory response induced by a high-fat diet in Wistar rats. Long-term HFD is an unreasonable dietary structure and thus increases ROS generation through fatty acid oxidation in the mitochondria of the liver but *via* No_*x*_ in the adipose tissue, and an oxidative stress may occur.^15^ This may induce inflammatory responses as well. Although oxidation processes are indispensable to life for energy and metabolism, excess ROS can lead to the damage of proteins, nucleic acids and carbohydrates of cells *in vivo*,^16^ and it causes a great threat to the health of the body, especially for patients with obesity. Various biomarkers of oxidative stress have been developed. Darwish *et al.*^17^ studied the changes in the MDA and T-AOC levels of liver in rats for evaluating the oxidative injury of alcohol and the alleviation of camel milk. Other studies suggest that antioxidant enzymes such as SOD, CAT and GSH-Px can also be used as indexes of oxidative damages.

MDA is a by-product produced by free radical-mediated lipid peroxidation, and its level is considered a good biomarker of oxidative stress.^18^ An elevated level of MDA is the result of imbalances in the redox state. In this study, significantly decreased levels of MDA in serum, liver and adipose were observed with different doses of bacterial suspension groups and fermented milk groups of *L. fermentum* DALI02 as compared to the model group. Particularly, the serum and adipose MDA levels of high dose bacterial suspension groups and fermented milk groups recovered to normal levels, with adipose MDA levels of high dose being even lower than normal level. These results indicated that *L. fermentum* DALI02 treatments could reduce oxidative stress to some degree in rats, especially higher doses had a complete protective effect on the serum and adipose of rats with the aspect of lipid peroxidation. The reduction effect of *L. fermentum* DALI02 on adipose MDA levels was obviously dose-dependent.

T-AOC depicts the synergistic interaction among different antioxidants, thus providing an insight into the delicate *in vivo* balance between oxidants and non-enzymatic antioxidants.^19^ HFD caused an elevation in the level of MDA with a significant reduction in T-AOC. We observed that despite the liver and adipose T-AOC levels being below the normal level, they were significantly higher in the model group, and also showed a slight increase with a dose-upward trend through different doses of probiotic treatment. The serum T-AOC levels in high-dose groups were even significantly higher than the normal levels and showed a dose-dependent effect in the serum. This indicated that *L. fermentum* DALI02 could improve the antioxidant defense system of hyperlipidemic rats.

As one of key scavengers of ROS, SOD can also protect host cells from an oxidative damage.^20^ Long-term HFD could induce a significant decrease in the SOD activity in liver tissue. Most lactic acid bacteria could produce SOD to dismutate superoxide radicals to oxygen and hydrogen peroxide, and its specific activity can not be influenced by the aerobic environment. A significantly higher liver SOD activity was observed in the probiotic treatment group as compared to that in the model group; especially, fermented milk groups were better with the normal level, indicating that the SOD production by *L. fermentum* DALI02 could keep scavenging free radicals at a normal level to promote the health of hyperlipidemic rats.

As another important antioxidant enzyme, GSH-Px is a glutathione-utilizing peroxidase and plays an important role in protecting organelles from oxidative injury.^14^ HFD could induce a significant decrease in the GSH-Px activity. Similar to the SOD activity mentioned above, *L. fermentum* DALI02 exerted a health-promoting effect on hyperlipidemic rats. Moreover, the different dose supplementation of *L. fermentum* DALI02 also showed a full recovery effect on the GSH-Px activity in liver. In addition, the liver GSH-Px activity almost recovered to normal levels through high doses of *L. fermentum* DALI02. Related reports have confirmed that GSH-Px activities increased *in vivo* after dietary supplementation with *L. fermentum* and *L. plantarum*.^21,22^ However, the hepatic GSH-Px activity did not show a dose-dependent effect, and there was no difference between the GSH-Px activity in medium and low dose groups. In comparison with the hepatic GSH-Px activity, the antioxidative capability of *L. fermentum* DALI02 was less effective with the adipose.

Similar to the effect of GSH-Px, CAT is also an important antioxidant enzyme and plays an important role in the catabolism of hydrogen peroxide *in vivo*.^21^ Similar to the GSH-Px activity as aforementioned, sera CAT activity did not show a dose-dependent effect and there was no difference between CAT activity in the medium and low dose groups.

Further, adipose tissue is not only actively involved in energy balance, but also an important organ to produce various cytokines that are involved in inflammatory pathways, such as TNF-α, IL-6, ADPN and resistin.^23,24^ The adipose gene expression of proinflammatory (TNF-α, IL-6) cytokines is elevated with obesity,^23^ whereas the expression levels of ADPN are lower in obese individuals.^25^ Furthermore, there is a close link among obesity, a state of chronic low-level inflammation and oxidative stress. Moreover, the oxidative stress can promote the dysregulation of adipocytokines.^26^ In an attempt to elucidate how *L. fermentum* DALI02 exerts its protective effects on the inflammatory response in hyperlipidemic rats, we further examined the levels of TNF-α, IL-6, ADPN and resistin in adipose tissue. TNF-α and IL-6 play important roles in the HFD-induced inflammation.^25,27^ ADPN is a protein that is predominantly produced by adipose tissue,^28^ and is one of the most abundantly expressed adipokines that plays a key role in inflammatory reactions and metabolic disturbances.^29^ ADPN treatment could suppress the hepatic production of TNF-α and plasma concentrations of this proinflammatory cytokine.^30^ Resistin is an adipocyte-secreted hormone that impairs glucose homeostasis and insulin action.^24^ The results of this study suggested that the levels of TNF-α, IL-6 and resistin of hyperlipidemic rats in adipose were significantly reduced with the supplementation of *L. fermentum* DALI02, whereas the ADPN level increased. This improved effect could be related with the enhanced antioxidant capacity against oxidative stress by supplementation of *L. fermentum* DALI02, so that the body can maintain a high level of ADPN, and lower the expression of TNF-α, IL-6 and resistin.

Our findings revealed that the antioxidative capability of cell lysates was less effective in comparison with the bacteria suspension and fermented milk of *L. fermentum* DALI02, for there was no significant difference between the liver MDA level in the cell lysate group and model group. Though CAT, T-AOC, SOD and GSH-Px activities were higher than the model group, they were less with different doses of bacteria suspension group and fermented milk group; similar results were also reflected with adipocytokines. One reason could be that the antioxidant enzymes and other substances were greatly damaged because of low pH, high presence of bile salts, proteases and other factors when cell lysates through the gastrointestinal environment, although the intracellular antioxidant enzymes and other antioxidant substances could be released by broken cells. Our previous studies have confirmed that the tolerance of *L. fermentum* DALI02 to artificial gastric juice, artificial intestinal fluid and bile salts were excellent, and thereby it could go through the gastrointestinal into the intestine and play an important role in reducing OS and inflammatory response. Therefore, the high-dose bacteria suspension and fermented milk can significantly alleviate the oxidative stress and inflammation in hyperlipidemic rats.

## 5 Conclusions

Results of this study showed that the supplementation of *L. fermentum* DALI02 improved the antioxidant status of hyperlipidemic rats significantly through the antioxidant indexes such as MDA levels, T-AOC, SOD, GSH-Px and CAT activities. Further, the improvement in serum seemed to be more effective than in liver and adipose, and was dose-dependent. Moreover, *L. fermentum* DALI02 could improve inflammation in hyperlipidemic rats by reducing the TNF-α, IL-6, and resistin levels and significantly increasing the level of ADPN. This study suggests that supplementation of foods containing *L. fermentum* DALI02 could be used in future to alleviate oxidative stress and inflammation, thereby contribute to the protective benefits of hyperlipidemia.

## Conflicts of interest

The authors declare that they have no conflict of interest.

## Supplementary Material
